# First right lobe living-donor hepatectomy after sleeve gastrectomy

**DOI:** 10.1186/s12893-018-0366-7

**Published:** 2018-05-29

**Authors:** Aiman Obed, Abdalla Bashir, Anwar Jarrad

**Affiliations:** 1 Hepatology, Gastroenterology and Hepatobiliary/Transplant Unit, Jordan Hospital, Amman, Jordan; 2 General and Transplant Surgery, Jordan Hospital, Amman, Jordan; 3 Hepatobiliary and Transplant Surgery, Jordan Hospital, Amman, Jordan

**Keywords:** First donor, NASH, Liver transplant, Living liver donation, Sleeve gastrectomy

## Abstract

**Background:**

Obesity presents one of the leading causes of many chronic liver disorders and injuries. Nowadays, non-alcoholic steatohepatitis (NASH) demonstrates a challenging issue for the global health system. NASH can progress to life-threatening conditions such as cirrhosis and hepatocellular or cholangio carcinoma. Currently, NASH cirrhosis is a major indication for liver transplant (LT).

**Case presentation:**

We present the case of a 37 year-old male who has lost 74 kg after undergoing successful laparoscopic sleeve gastrectomy (SG) four years ago. Recently, he underwent right hepatectomy in the course of living-donor liver transplantation for his sick father in our clinic. Before the SG was conducted four years ago, his weight was at 157 kg and his Body Mass Index (BMI) at 49 kg/m^2^. At that time, Ultrasound examination showed severe fatty liver changes and intraoperative inspection of the liver was consistent with that observation. At the time of surgery, he weighed 83 kg and his BMI was at 27 kg/m^2^. An effective weight reduction after bariatric surgery might protect NASH patients from further deterioration of their medical condition.

**Conclusion:**

To our knowledge, we report the first successful case of a right lobe living-donor hepatectomy in a patient who previously underwent successful laparoscopic sleeve gastrectomy (LSG).

## Background

Obesity is associated with many chronic diseases including non-alcoholic fatty liver disease (NAFLD) and non-alcoholic steatohepatitis (NASH). Untreated NASH can progress to fibrosis and cirrhosis. Furthermore, NASH can increase the risk of developing cholangio carcinoma and hepatocellular carcinoma. NASH cirrhosis is becoming a major indication for liver transplant (LT) [1–3].

Weight reduction by bariatric surgery has shown to be an effective way of preventing the progression of NAFLD to chronic liver disease and cirrhosis [4–6]. Additionally, bariatric surgery was found to be feasible and safe in selected cirrhotic patients, liver transplanted patients and in the setting of combined procedures such as liver transplantation [7–10].

Significant weight loss after SG might prevent NASH patients from further deterioration of their liver function.

Herein we report the first right lobe living-donor hepatectomy four years after successful laparoscopic sleeve gastrectomy in a 37 year-old man.

## Case presentation

A 37 year-old male who underwent successful laparoscopic sleeve gastrectomy four years ago and who has consequently lost 74 kg was presented to our clinic with other family members willing to provide their sick father with a right lobe for the liver transplantation. Before the SG was done four years ago his weight was at 157 kg and his Body Mass Index (BMI) at 49 kg/m^2^. At that time Ultrasound examination showed severe fatty liver changes with diffuse increased hepatic parenchymal echogenicity and also intraoperative inspection of the liver was consistent with that observation. Unfortunately, liver biopsy was not taken. We did not quantify steatosis with other imaging modalities, such as MRI or CT, since the liver donation was not an issue at that time.

At the time of surgery, he weighed 83 kg and his BMI was at 27 kg/m^2^. His lowest weight after SG was at 80 kg.

Full donor work up including liver biopsy was completed. The liver biopsy showed steatosis in less than 5%, no inflammation, no hepatocyte ballooning and no fibrosis.

He was chosen as an appropriate donor with a total liver volume of 1800 cm^3^ and a future remnant liver volume of 900 cm^3^. The graft-to-recipient weight ratio (GRWR) was at 0.9%.

Donor work up was completed including extended lab analysis, anesthesia, and cardiac, assessment, psychiatric team and ethical committee evaluation for organ transplantation and was approved for liver donation.

The Liver Transplant Board confirmed the indication for liver transplant and approved the living donor liver transplantation (LDLT). After completing the evaluation, LDLT was performed.

His father suffered from end-stage liver disease secondary to NASH cirrhosis, accompanied by type 2 diabetes mellitus. Evaluations revealed a Child-Pugh stage B, a model for end-stage liver disease (MELD) calculated score of 17 and a body weight at 100 kg.

**Fig. 1:**
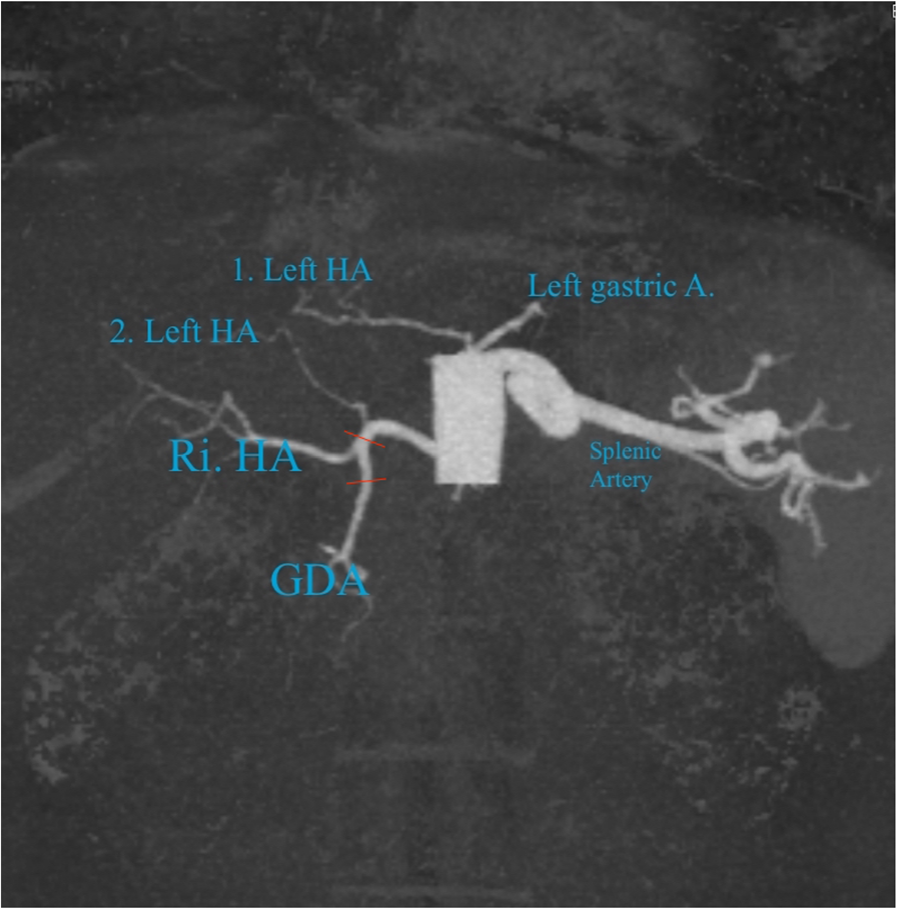
Preoperative liver triple phase CT- scan showed right hepatic artery arising from celiac trunk with relevant left hepatic artery arising from left gastric artery; short red lines mark the split lines of common hepatic artery and gastroduodenal artery coming out with the right liver lobe

During the right lobe hepatectomy, only minimal adhesions were found. The liver appeared grossly normal with a sharp left lateral lobe. The left liver lobe was supplied by a branch of the left gastric artery and a smaller branch of the main hepatic artery. The donor common hepatic artery, after the bifurcation of the left HA branch, was recovered with a segment of the gastroduodenal artery (Fig. 1). We chose to perform a right lobe donation, the most commonly performed procedure in our center, since it provides a sufficient liver remnant volume. Additionally, in this particular case the left lobe had two small arteries in comparison with the right lobe that only had a single big right hepatic artery. Also, there were no veins from the right lobe merging into the middle hepatic vein.

**Fig. 2:**
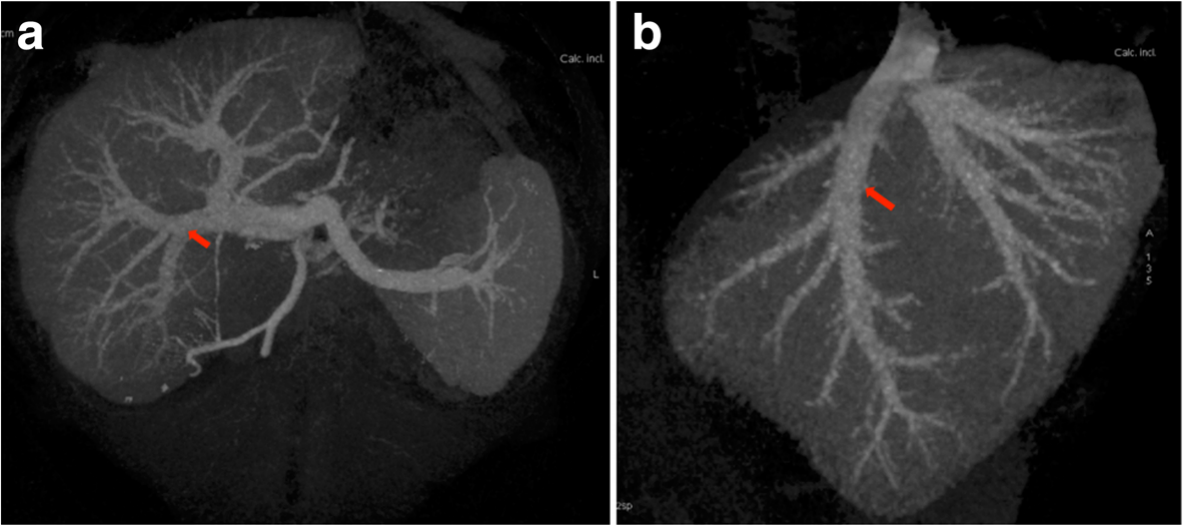
Preoperative liver triple phase CT-scan showed normal appearance of right hepatic vein (**a**) and right portal vein (**b**)

On the recipient side, left and right hepatic arteries were divided and a large anastomosis with branch-patch reconstruction was established. The right hepatic vein and the right portal vein appeared without any abnormalities (Fig. 2). The donor operation time was 3 h and 20 min. The donor didn’t receive any blood products and was admitted to our intensive care unit for one day.

The donor had an uneventful recovery. On the first postoperative day, patient ambulation and clear fluid oral intake took place and he was discharged after seven days with normal liver function (Table 1).

**Table 1:** Shows postoperative liver function test for seven days

Post-operative day	AST (IU/L)	ALT (IU/L)	Total Bilirubin (mg/dL)
0	159	194	1.4
1	211	178	2.6
2	170	134	1.9
3	100	98	1.7
4	79	88	1.7
5	52	46	1.5
6	43	34	1.3
7	33	29	1.1

Currently, eight months after the right lobe donation procedure, his liver function appears to be normal and his body weight remains stable in comparison to his weight prior to surgery (BMI = 27 kg/m^2^).

Graft reperfusion in the recipient was normal and liver biopsy was not required.

Similarly, the recipient had an uneventful postoperative course. Ambulation and reintroduction of oral feeding were started on postoperative day two and he was discharged with normal liver function three weeks after LDLT. His diabetes disappeared after three months.

## Discussion and conclusions

Obesity has reached an epidemic level. In many countries the incidence of obesity, defined by a BMI ≥ 35 kg/m^2^, ranges between 7 and 24% and has even higher prevalences in Europe [11]. It is one of the main contributors to the global burden of the chronic clinical spectrum, including NAFLD. NAFLD includes a variety of liver injuries that range from simple steatosis to non-alcoholic steatohepatitis (NASH) [12].

Weight reduction by bariatric surgery has proved to be an effective way of preventing the progression of NAFLD to chronic liver disease and cirrhosis [4–6]. NASH cirrhosis presents the end stage of the spectrum of NAFLD and is becoming a main indication for LT in the United States. Presently, it is the third most common indication for LT and it is estimated to become the most common indication for LT within the next 1–2 decades.

Bariatric surgery was found to be convincing and safe in selected cirrhotic patients, liver transplanted patients and in the setting of combined procedures such as liver transplantation.

A good percentage of obese patients are disqualified at the time of presentation for LT due to the high risk of combined diseases.

Obesity is certainly associated with diabetes mellitus, coronary disease and tumors, and might imply significant morbidity and mortality post-LT. There are several surgical strategies to improve the outcome in this group of patients, including bariatric surgery before LT, bariatric surgery after LT and bariatric surgery [13–19] at the time of LT as initially described by Heimbach et al., who reported their expertise of combined LT and gastric sleeve resection (SG) in seven patients with a BMI greater than 35 kg/m^2^ [10].

The advantages of combined LT with SG are certainly the reduction to one operative implementation for the patient and the consecutive prevention of a second operation with potentially severe adhesions. Recent studies have demonstrated a significant improvement of NAFLD Activity Score and fibrosis after SG [20].

Significant weight loss after bariatric surgery could help NASH patients to avoid the progression that might require liver transplant.

Even in the absence of published solid data, liver donation after major surgery seems to be feasible. Patients who are willing to donate their liver after successful bariatric surgery should be evaluated. If the donor evaluation reveals no medical contraindication, LDLT could be performed; probably with the same risks that donors without SG in their history would have.

In general, all treatments that lead to the increase of the donor pool are welcomed. In absence of the gastric sleeve procedure, our patient would not have been eligible for donation.

The lack of donor biopsy at the time of sleeve gastrectomy demonstrates a study limitation in our case.

To our knowledge, we report the first successful case of a right lobe living-donor hepatectomy in a patient who previously underwent successful laparoscopic sleeve gastrectomy (LSG).

In conclusion, donor hepatectomy and LDLT appear to be safe and feasible operations from donors who underwent SG for obesity. Larger studies are needed to confirm this strategy, especially in the light of donor shortage in the field of liver transplantation.

## Abbreviations

BMI: Body Mass Index
GRWR: Graft-to-recipient weight ratio
LDLT: Living donor liver transplant
LSG: Laparoscopic sleeve gastrectomy
LT: Liver transplant
NAFLD: Non-alcoholic fatty liver disease
NASH: Non-alcoholic steatohepatitis
SG: Sleeve gastrectomy
